# Acceptance of a COVID-19 Vaccine Before it is Available in China During the Pandemic

**DOI:** 10.3389/ijph.2021.1604092

**Published:** 2021-10-25

**Authors:** Jian Xu, Cong Liu

**Affiliations:** ^1^ School of Media and Communication, Shanghai Jiao Tong University, Shanghai, China; ^2^ China Institute for Urban Governance, Shanghai Jiao Tong University, Shanghai, China; ^3^ Institute of Cultural Innovation and Youth Development, Shanghai Jiao Tong University, Shanghai, China

**Keywords:** COVID-19, pandemic, vaccine, China, acceptance

## Abstract

**Objectives:** China was believed to be the country with the world’s highest acceptance rate of the COVID-19 vaccine following several investigations. This study aims to explore the Chinese acceptance of a COVID-19 vaccine before it is made available, including its determinants.

**Methods:** A cross-national online survey was conducted covering all 31 provinces of mainland China. The survey consists of the demographic variables, acceptance of a self-paid COVID-19 vaccine as the dependent variable, and the 3Cs factors (i.e., confidence, convenience, and complacency) as the independent variables.

**Results:** Among the 1,532 participants, 57.9% accepted to get a self-paid COVID-19 vaccine. COVID-19 vaccine acceptors were more likely to be concerned about the effectiveness of the vaccines, believe that they were at risk of COVID-19 infection, have a high perceived susceptibility of COVID-19, and trust in the health care system.

**Conclusion:** Findings indicate that the critical task in the early stage of the COVID-19 vaccine development in China is to increase the tolerance to some intuitive concerns about the vaccines, put more emphasis on the communication of the saliency of the disease threats, and effectively translate people’s trust in the government into vaccine acceptance.

## Introduction

The COVID-19 (Coronavirus disease 2019) remains rampant [1]. In some countries, there have been second- or even third-wave outbreaks [2, 3]. In response to the outbreaks, most countries have taken measures to block transit, restrict entry and exit, and impose lockdowns in some cities or regions. China’s epidemic trend is somewhat under control, but there are still occasional small-scale outbreaks with the arrival of autumn and winter [4].

The director of the World Health Organization (WHO) claimed that the most efficient way to bring this crisis to an end is with a safe and effective vaccine, manufactured in large quantities and distributed globally [5]. There are currently more than 50 COVID-19 vaccine candidates being trialed [6]. A global survey on attitudes towards a COVID-19 vaccine conducted by the World Economic Forum in July 2020 revealed that China reported the highest willingness to vaccinate (97%) compared to the other 26 countries—far higher than the world average (74%) [7]. Another global survey of COVID-19 vaccine acceptance conducted in 19 countries in June 2020 also showed that China gave the most positive responses (88.6%) compared to the world average of 71.5% [8]. However, results from two Chinese survey studies conducted in March and May 2021, respectively, were far less optimistic. In one study, only 52.5% of the participants wanted to get vaccinated as soon as possible, while 47.7% would delay the vaccination until the safety was confirmed [9]. In the other study, only 28.7% of the participants responded “definitely yes” for their intentions to receive a COVID-19 vaccine [10].

It was also claimed that in most of the countries surveyed, current levels of acceptance of a COVID-19 vaccine are insufficient to meet the requirements for herd immunity [8]. Based on the COVID-19 R0 estimation of 5.7 in China, the threshold for combined vaccine efficacy and herd immunity needed for disease extinction is 82% [11]. In other words, only when more than 82% of the Chinese population are vaccinated (or naturally infected) can herd immunity be achieved. This rate for the United States was estimated to be 75–80% [12]. Besides, numerous problems and adverse reactions have continuously been exposed in the process of vaccine development in different countries [13–15]. Since people were found to be more hesitant about vaccination following negative vaccine incidents [16–19], it is unclear how the public’s acceptance of the COVID-19 vaccine will be further affected in the current circumstance.

The WHO listed vaccine hesitancy as one of the top 10 threats to global health in 2019 [20]. Vaccination hesitancy refers to delay in acceptance or refusal of vaccines despite the availability of vaccine services [21]. Vaccine hesitancy is an increasingly recognized global problem that varies between and within countries, and it is especially likely to happen with newly introduced vaccines [22]. Vaccine hesitancy is not driven by a simple set of individual factors. The Confidence, Convenience, and Complacency (3Cs) model was used for grasping factors of vaccine hesitancy by the researchers [16, 23, 24]. Confidence is defined as the trust in the safeness and effectiveness of vaccines; the system that delivers them, including the health services and health professionals; and the policymakers. Numerous studies have found that the safeness and effectiveness of a vaccine positively influence people’s acceptance of the vaccine, especially for a newly developed vaccine such as the COVID-19 vaccine [25–28]. Confidence in the vaccine delivery system (e.g., the Food and Drug Administration) and trust in government were associated with a lower vaccine hesitancy and higher acceptance [8, 16, 29, 30]. Convenience is usually measured by the extent of physical availability, affordability, willingness-to-pay, and so on. For example, the price of the vaccines was found to be a factor that influence the acceptability and willingness-to-pay for the vaccines [31, 32]. Fully covered health insurance was also found to be a factor promoting participant’s vaccine acceptability [22]. Complacency exists where perceived risks of vaccine-preventable diseases are low and vaccination is not deemed a necessary preventive action [22, 25]. Perceived susceptibility was found to be positively related to the willingness-to-pay for the hepatitis B vaccine in a Malaysian study [33]. The perceived likelihood of getting COVID-19 infection was found to be related to higher COVID-19 vaccine acceptability among American samples [28]. Besides the 3Cs variables, demographic characteristics such as gender, age, household income, education, and so on also influence the acceptance of vaccines against various diseases and across nations [8, 28, 34].

The WHO claimed that each country should develop a strategy to increase acceptance and demand for vaccination and reinforced the importance of understanding the extent and nature of hesitancy at a local level, in specific contexts, and on a continuing basis [22]. Although previous global surveys provide valuable information for the comparisons of the relative vaccine acceptance for different countries, it is difficult to accurately capture the actual level of vaccine acceptance in each country due to the limitation of the sample representativeness. It remains to be seen whether the situation of vaccine acceptance among a more representative Chinese sample is as optimistic as reported. The over-optimistic survey results will obviously not be conducive to the effective distribution of the vaccine by the government. Besides, in sharp contrast to the first half of the year, the COVID-19 epidemic in China is well under control, the life of people has returned to the right track, and the vaccine research and development has also made great progress in the second half of the year. In this context, the Chinese acceptance of the COVID-19 vaccine as well as its determinants is worth further investigating.

## Methods

### Study Design

The data were collected online by a sample service provider (i.e., Changsha Ranxing IT Ltd.) that owns one of the biggest online samples with more than 2.6 million members all over China. The sample aimed to cover all the 31 provinces of mainland China with no less than 30 respondents from each province. The number of respondents from each of the province-level municipalities and the first-tier cities (i.e., Beijing, Shanghai, Tianjin, Chongqing, Guangzhou from Guangdong Province, and Shenzhen from Guangdong Province), as well as the city with the highest accumulated cases (i.e., Wuhan), were particularly set to be no less than 100 respectively. The survey was conducted by randomly inviting 7,622 members *via* webpages and Wechat from September to October 2020. A total of 1,797 members responded to the invitation, among them, 265 invalid responses were systematically and manually eliminated by the sample service provider, and the final valid responses received were 1,532 with a response rate of 20.1%. Odds ratios of the predictors of the COVID-19 vaccine acceptance obtained from previous studies indicated small to medium effect sizes [8, 9], and the sample size of 1,532 was able to obtain a power of 75–80% even with the smallest effect size d of 0.1 for a two-tailed alpha of 0.05 [35].

Weight calibration is commonly used to correct for nonresponse and coverage errors in social, behavioral, health, and other surveys [36, 37]. The proportional fitting calibration is one of the most commonly implemented methods in which weight calibration adjusts the survey weights so that the weighted totals (means, proportions) agree with the externally known benchmarks [38, 39]. To make the sample composition of this study best reflect the demographic profile of the 18–65 population in mainland China, the data were weighted based on the proportions of age (i.e., 18–30, 31–40, 41–50, 51–60, and 61–65) and gender (i.e., male and female) groups in the most recent census data^1^ [40].

### Measurements

#### Dependent Variable

Acceptance of a COVID-19 vaccine was measured by a 3-point scaled asking “are you going to uptake the COVID-19 vaccine at your own expense when it is available”? (1 = “no”, 2 = “not sure”, 3 = “yes”). This variable was further combined into two categories (0 = “no” or “not sure”, 1 = “yes”) as the binary dependent variable of COVID-19 vaccine acceptance.

#### 3Cs Model Variables

The 3Cs (i.e., confidence, convenience, and complacency) variables include both dichotomous variables and continuous variables. Confidence variables include three dichotomous indicators of “I’m concerned about the side effects”, “I’m concerned about its effectiveness” and “I’m against vaccine in general” (0 = “no”, 1 = “yes”); and two continuous indicators of trust in the health system (4 items; 5-point response scale ranging from “totally disagree” to “totally agree”) and trust in government responses to COVID-19 (3 items; 5-point response scale ranging from “totally disagree” to “totally agree”). Convenience variables include two dichotomous indicators of “I worry the price will be too high” and “I don’t have the time” (0 = “no”, 1 = “yes”). Complacency variables include two dichotomous indicators of “I’m not enough at risk from COVID-19” and “I don’t need to be vaccinated if most people are vaccinated” (0 = “no”, 1 = “yes”); and a continuous indicator of perceived susceptibility of COVID-19 (six items; 5-point response scale ranging from “totally disagree” to “totally agree”).

#### Demographic Variables

Participants were asked about their demographic information including gender, age, education level, socio-economic status, and health condition.

### Statistical Analysis

Data were analyzed using SPSS version 25.0 (SPSS, Chicago, IL, United States). Descriptive statistics (i.e., frequencies and percentages for dichotomous variables, means, and standard deviations for continuous variables) were calculated. A logistic regression model was employed to identify determinants of participant’s acceptance of a COVID-19 vaccine. In the first step, univariable analyses using simple logistic regressions were conducted. In the second step, all variables with *p* < 0.2 in the first step were included in the multivariable analysis using multiple logistic regression. The crude odds ratio (OR), adjusted odds ratio, and 95% confidence intervals (CIs) were used as indicators of the strength of association. A *p*-value of 0.05 was used as the level for statistical significance.

## Results

### Demographic Characteristics

Just over half of the participants were male (51%) and most participants were from the 18–30 (30.8%) and 31–40 (23.8%) age group, had some college or college education (80.6%), reported an average social economic status (49.3%) and a good health condition (62.9%). See Table 1 for more details.

**TABLE 1:** Acceptance of a COVID-19 vaccine by demographic characteristics and dichotomous confidence, convenience, and complacency model variables in mainland China, 2020^a^.

	n (%) 1,532	Acceptors n (%)
Demographic characteristics		
Gender		
Female	750 (49.0)	421 (56.1)
Male	781 (51.0)	466 (59.7)
Age Group		
18–30	471 (30.8)	301 (63.9)
31–40	365 (23.8)	247 (67.7)
41–50	354 (23.1)	203 (57.2)
51–60	248 (16.2)	120 (48.2)
61–65	93 (6.0)	16 (17.4)
Education Level		
Less than high school	42 (2.7)	7 (16.7)
High school	112 (7.3)	65 (58.0)
Some college/college	1235 (80.6)	726 (58.8)
Post-graduate	143 (9.4)	90 (62.9)
Social Economic Status		
Low	593 (38.7)	293 (49.4)
Average	755 (49.3)	469 (62.2)
High	184 (12.0)	125 (67.9)
Health Condition		
Poor	78 (5.1)	37 (47.4)
Average	490 (32.0)	253 (51.6)
Good	964 (62.9)	597 (62.0)
3Cs Model Variables - Dichotomous		
Confidence – I’m concerned about the side effects		
No	272 (17.7)	139 (51.3)
Yes	1260 (82.3)	748 (59.4)
Confidence – I’m concerned about its effectiveness		
No	687 (44.9)	343 (49.9)
Yes	844 (55.1)	544 (64.5)
Confidence - I’m against vaccines in general		
No	1481 (96.7)	867 (58.5)
Yes	51 (3.3)	20 (39.2)
Convenience - I worry the price will be too high		
No	428 (27.9)	247 (57.7)
Yes	1104 (72.1)	640 (58.0)
Convenience - I don’t have the time		
No	1487 (97.1)	858 (57.7)
Yes	44 (2.9)	29 (65.9)
Complacency - I’m not enough at risk from COVID-19		
No	1354 (88.4)	831 (61.4)
Yes	178 (11.6)	56 (31.5)
Complacency - I don’t need to be vaccinated if most people are vaccinated		
No	1419 (92.6)	833 (58.7)
Yes	113 (7.4)	54 (47.8)

a Frequencies and crosstabs in SPSS round the sums of cell weights, affecting the marginal counts and the percentages slightly, therefore, some variables adding up to 1,531 while others adding up to 1,532.

### Acceptance of a COVID-19 Vaccine

Overall, 57.9% (887/1532) of the participants accepted getting a self-paid COVID-19 vaccine, and 42.1% (645/1532) were classified as vaccine hesitant (7.2% rejected and 34.9% were not sure). The concerns about COVID-19 vaccines (Table 1) for most of the participants were side effects (82.3%), the price would be too high (72.1%), and effectiveness (55.1%), while a small portion of the participants were against vaccination in general (3.3%), thought they did not have the time (2.9%), thought they were not enough at risk (11.6%), or thought they did not need to be vaccinated if most people were vaccinated (7.4%). For those who accepted the COVID-19 vaccine (Table 2), the means of perceived susceptibility of COVID-19, trust in the health system, and trust in government responses to COVID-19 were 3.90 (SD = 0.65), 4.17 (SD = 0.52), and 4.51 (SD = 0.53), respectively. In contrast, for the vaccine hesitant, the means of susceptibility of COVID-19, trust in the health system, and trust in government responses to COVID-19 were 3.31 (SD = 0.77), 3.87 (SD = 0.63), and 4.33 (SD = 0.74), respectively.

**TABLE 2:** Acceptance of a COVID-19 vaccine by continuous confidence, convenience, and complacency model variables in mainland China, 2020.

	Mean (SD) hesitants *n* = 645	Mean (SD) acceptors *n* = 887
3Cs model variables - continuous		
Complacency - Perceived Susceptibility of COVID-19	2.91 (0.91)	3.64 (0.83)
I’m worried about being infected if I don’t get vaccinated	2.96 (1.12)	3.81 (1.06)
I’m worried about infecting people around me if I don’t get vaccinated	2.80 (1.22)	3.58 (1.20)
I would be infected if I don’t get vaccinated	2.89 (1.02)	3.55 (1.00)
People around me would be infected if I don’t get vaccinated	3.01 (1.10)	3.61 (1.07)
Confidence - Trust in Health System	3.87 (0.63)	4.17 (0.52)
I believe in the opinions and suggestions of experts and doctors	4.19 (0.72)	4.45 (0.77)
I believe that our local medical service has sufficient treatment capacity	3.99 (0.81)	4.27 (0.87)
If I get infected, I’m confident in the treatment	3.72 (0.96)	4.06 (1.05)
The medical system will protect the privacy of those who are infected	3.60 (0.91)	3.89 (0.93)
Confidence - Trust in government responses to COVID-19	4.33 (0.74)	4.51 (0.53)
I agree with the government’s measure of tracking people’s travel records	4.26 (0.83)	4.42 (0.90)
I agree with the government’s measure of collective quarantine for the close-contact or high-risk groups	4.34 (0.78)	4.55 (0.91)
I agree with the government’s measures to close down cities and communities during the epidemic	4.39 (0.75)	4.54 (0.84)

### Factors in Vaccination Decisions

The univariate analyses revealed that age, social economic status, health condition, and five of the dichotomous 3Cs variables and all three of the continuous 3Cs variables were significant. In multivariate analysis (Table 3), participants were more likely to accept the COVID-19 vaccine if they were being male (OR = 1.29, 95% CI: 1.00–1.65, *p* < 0.05), had high school education (OR = 4.24, 95% CI: 1.54–11.65, *p* < 0.01) or some college/college education (OR = 3.41, 95% CI: 1.34–8.70, *p* < 0.05) versus less than high school education, had an average (OR = 1.53, 95% CI: 1.16–2.01, *p* < 0.01) or high social economic status (OR = 2.30, 95% CI: 1.47–3.60, *p* < 0.001) versus low social economic status, concerned about the effectiveness of the vaccine (OR = 1.74, 95% CI: 1.36–2.22, *p* < 0.001), reported higher perceived susceptibility of COVID-19 (OR = 3.03, 95% CI: 2.51–3.65, *p* < 0.001), or reported higher trust in the health system (OR = 1.92, 95% CI: 1.50–2.47, *p* < 0.001). However, participants were less likely to accept the COVID-19 vaccine if they were at the 41–50 age group (OR = 0.72, 95% CI: 0.52–1.00, *p* = 0.05), the 51–60 age group (OR = 0.34, 95% CI: 0.23–0.49, *p* < 0.001) or the 60–65 age group (OR = 0.06, 95% CI: 0.03–0.12, *p* < 0.001) versus the 18–30 age group, or thought they were not enough at risk from COVID-19 (OR = 0.36, 95% CI: 0.24–0.54, *p* < 0.001).

**TABLE 3:** Univariable and multivariable correlates of acceptance of a COVID-19 vaccine in mainland China, 2020.

	Univariable		Multivariable	
	OR (95% CI)	*p*-value	OR (95% CI)	*p*-value
Demographic characteristics				
Gender		0.149		
Female	Ref		Ref	
Male	1.16 (0.95–1.42)	0.149	1.29 (1.00–1.65)*	0.046
Age Group		0.000		
18–30	Ref		Ref	
31–40	1.19 (0.89–1.59)	0.241	0.91 (0.65–1.27)	0.571
41–50	0.76 (0.57–1.01)	0.054	0.72 (0.52–1.00)*	0.050
51–60	0.53 (0.39–0.72)***	0.000	0.34 (0.23–0.49)***	0.000
61–65	0.12 (0.07–0.21)***	0.000	0.06 (0.03–0.12)***	0.000
Education Level		0.171		
Less than high school	Ref		Ref	
High school	6.95 (2.82–17.13)***	0.000	4.23 (1.54–11.65)**	0.005
Some college/college	7.21 (3.15–16.49)***	0.000	3.41 (1.34–8.70)*	0.010
Post-graduate	8.52 (3.51–20.68)***	0.000	2.62 (0.95–7.20)	0.062
Social Economic Status		0.000		
Low	Ref		Ref	
Average	1.68 (1.35–2.10)***	0.000	1.53 (1.16–2.01)**	0.003
High	2.16 (1.53–3.07)***	0.000	2.30 (1.47–3.60)***	0.000
Health Condition		0.000		
Poor	Ref		Ref	
Average	1.19 (0.74–1.92)	0.483	0.71 (0.38–1.33)	0.283
Good	1.82 (1.14–2.89)*	0.011	0.97 (0.52–1.79)	0.913
3Cs Model Variables - Binary				
Confidence - I’m concerned about the side effects		0.016		
No	Ref		Ref	
Yes	1.38 (1.06–1.80)*	0.016	0.75 (0.54–1.05)	0.090
Confidence - I’m concerned about its effectiveness		0.000		
No	Ref		Ref	
Yes	1.82 (1.48–2.24)***	0.000	1.74 (1.36–2.22)***	0.000
Confidence - I’m against vaccines in general		0.008		
No	Ref		Ref	
Yes	0.47 (0.27–0.83)**	0.009	1.13 (0.53–2.42)	0.752
Convenience - I worry the price will be too high		0.937		
No	Ref			
Yes	1.01 (0.81–1.27)	0.937		
Convenience - I don’t have the time		0.263		
No	Ref			
Yes	1.43 (0.76–2.68)	0.271		
Complacency - I’m not enough at risk from COVID-19		0.000		
No	Ref		Ref	
Yes	0.28 (0.21–0.40)***	0.000	0.36 (0.24–0.54)***	0.000
Complacency - I don’t need to be vaccinated if most people are vaccinated		0.027		
No	Ref		Ref	
Yes	0.65 (0.44–0.95)*	0.027	0.68 (0.42–1.11)	0.127
3Cs Model Variables - Continuous				
Complacency - Perceived Susceptibility of COVID-19	3.24 (2.75–3.82)***	0.000	3.03 (2.51–3.65)***	0.000
Confidence - Trust in Health System	2.48 (2.05–3.00)***	0.000	1.92 (1.50–2.47)***	0.000
Confidence - Trust in government responses to COVID-19	1.55 (1.32–1.83)***	0.000	0.89 (0.70–1.13)	0.334

*p < 0.05.

**p < 0.01.

***p < 0.001.

CI, Confidence Interval; Ref, reference.

## Discussion

This study explored how the 3Cs factors influence people’s acceptance of a COVID-19 vaccine among the Chinese sample that reflects the demographic profile of the 18–65 population in mainland China. Results showed that only 57.9% of the Chinese population accept a COVID-19 vaccine. This rate is similar to another Chinese survey conducted in early 2020 [9] but is far lower than that reported in the global surveys [7, 8]. It is worth noticing that there is still a long way to achieving the goal of community immunity in China or in the world. Among the surveyed Chinese sample, 70.5% had to get self-paid vaccines before, and this implies that the anti-vaccines are only the minority and there is space for improving the acceptance for the COVID-19 vaccines. This survey also showed that side effects (82.3%), effectiveness (55.1%), and price (72.1%) are the top concerns about the COVID-19 vaccines, although not all these factors influence the acceptance. COVID-19 vaccine acceptors are more likely to be male and below 40 years of age, have a high school or college education and average or high socio-economic status, be concerned about the effectiveness of the vaccines, believe that they were at risk of COVID-19 infection, have a high perceived susceptibility of COVID-19, and trust in the health care system. Most of these findings were supported by previous studies [9, 10, 28].

Risk factors among the demographic variables for vaccine acceptance were found to be female gender, ages of above 40 years, lower education or post-graduate education, and low socio-economic status. A few other studies conducted in the same period in China also indicated that vaccination hesitancy was more prevalent among the older adults [10, 41]. This could be due to the specific vaccination policy in China as well as the media effects. For example, those above 60 years of age (and teenagers below 18) were excluded for the first batch of vaccination considering the uncertain risk factors among them to uptake the newly developed vaccine. Besides, information sources on COVID-19, including social media, other internet/webpages, and family/friends could also increase the negative vaccine intent of the older adults [42]. Findings of socio-economic status coincide with past research, which found that acceptance of vaccines was lower among individuals with lower socioeconomic status or without health insurance [16, 25]. This indicates that if the COVID-19 vaccines can be provided free of charge or at the least a lower cost in China as well as in other countries, the vaccine hesitancy due to financial barriers will be greatly reduced, especially among the low- and middle-income populations. Future vaccine communication strategies should consider the characteristics, scientific and health literacy of the subpopulations with higher vaccination hesitancy, and identify their trust sources of information or recommendation [8, 43].

Analyses among the 3Cs variables indicated that confidence is a critical factor that influences the acceptance of a COVID-19 vaccine, nevertheless, lower confidence does not have a merely negative influence. First, it is worth noting that concerns about vaccines do not necessarily mean lower acceptance. In contrast, people who were concerned about the effectiveness of the COVID-19 vaccines were more likely to get vaccinated. Similarly, people were highly concerned about the side effects, but it did not become a barrier for COVID-19 vaccination, as was indicated by this study. In other words, particular concerns, such as the effectiveness, could be reflections of attention and rational thoughts about the vaccine, or even signs of acceptance to the vaccine, especially given that the COVID-19 vaccines are newly developed in a very limited time. Second, perceived trustworthiness of the health care system during the COVID-19, including suggestions from doctors and experts, treatment capacity of the local health care system, and privacy protection to the patients were found to be a key factor that enhances people’s acceptance of the COVID-19 vaccine. Third, trust in government responses was not found to affect the acceptance of the COVID-19 vaccine. This suggests that government measures against the pandemic were not effectively translated into confidence in the vaccines. This could be determined by the special situation in China during the pandemic. The epidemic in China was rapidly controlled by the collective actions of the people under the directions of the government long before the vaccines were developed. This means people tend to believe more in the government measures against the disease rather than rely on vaccines [8]. Complacency variables including belief in their likelihood of COVID-19 infection and perceived susceptibility of the disease were found to positively increase the acceptance to a COVID-19 vaccine. Many previous studies regarding vaccine acceptance had found similar evidence supporting the influence of the complacency variables [9, 28]. This could be explained by the theories regarding the saliency of the disease threat, which was found to be a promising factor that reduces vaccine hesitancy [44, 45]. Convenience factors including the price of the vaccine and time availability were not among the key factors influencing the acceptance of the vaccine among the Chinese sample. Although many people had concerns about the price, but it did not become a determinant for the acceptance of vaccines either in the univariate analysis or in the multivariate analysis, and it is the same with the time issue. Similarly to the side effect and effectiveness concern, this result also reflects that the price issue could be a rational concern instead of a barrier to one’s acceptance of a vaccine.

Findings of the current study provide insights into the communication strategies dealing with vaccine hesitancy. First, fighting against the anti-vaccine messages and promoting the safeness and effectiveness of the COVID-19 vaccines might not be the only solution to vaccine hesitancy. It is important to realize that certain concerns could be rational and intuitive reactions to a newly developed vaccine, not only for those that are vaccine hesitant, but also for those who are willing to get vaccinated. According to the cultural attraction theory, vaccination is a counter-intuitive action in nature, therefore, pro-vaccination messages are more difficult to spread compared to the more intuitive anti-vaccination messages such as information about the vaccines’ side effects and low effectiveness [45, 46]. Therefore, it is important to tolerate rational concerns about the vaccines on one hand and make the pro-vaccination messages to be as intuitive as possible on the other. Furthermore, emphasis could be put on the risks and susceptibility of the disease so as the enhance the saliency of the threat of disease versus the saliency of the harm of vaccines.

Finally, it is of vital significance to find solutions to the unique problems in each nation, instead of repeatedly emphasizing the communication strategies on trust-building and fighting against those rational concerns. As was mentioned above, concerns about vaccines are intuitive and trust in the health care system and the government during this crisis are very high among the Chinese people—these are not the most urgent problems relevant to vaccine hesitancy in China. A unique phenomenon in China indicated by this study was that the high level of trust in government responses was not effectively translated into vaccination acceptance. In such circumstances, it is important to persuade the public that fighting against the pandemic with quarantines and area shutdowns by the government will not be the only way out of this crisis in the long run, and the vaccination is.

This study has several limitations. First, although the sample covered all the 31 provinces in mainland China, the sample size of 1,532 could have limited representativeness of different ages, genders, incomes, and regional groups, and the weighted sample may still be more educated, more Internet active, and more urban than the general adult population since the data was collected online. Second, from Jan 2021, the Chinese government announced the provision of COVID-19 vaccines free of charge, which is 8 months after our survey. Therefore, in a similar way to all other cross-sectional studies, our results only serve as a snapshot of the acceptance rate at a certain point in time in the drastically changing situation of the COVID-19 pandemic and vaccine development. However, it is of greater importance to our study to explore the relatively more stable relationships between people’s vaccination acceptance and other attitudinal factors, so as to provide practical implications to the related parties.

### Conclusion

This study indicated a far lower acceptance rate of a COVID-19 vaccine in China compared to that reported in the global surveys. It also explored how acceptance of a COVID-19 vaccine will be influenced by the demographic characteristics and the confidence, convenience, and complacency factors among the Chinese sample in a period that the local epidemic was well under control. Findings indicated that besides the communication strategies targeting subpopulations, dealing with anti-vaccination messages, and engaging in trust-building, the more critical a task in the current stage of China it is to increase the tolerance to the intuitive and rational concerns about the newly developed COVID-19 vaccines, emphasize the saliency of the disease threats, as well as effectively translate people’s trust in government into vaccine acceptance.
